# Supplementary material for: Non-destructive test-based assessment of uniaxial compressive strength and elasticity modulus of intact carbonate rocks using stacking ensemble models
Source: PLoS One. 2024 Jun 10;19(6):e0302944. doi: 10.1371/journal.pone.0302944 (PMC11164374; doi:10.1371/journal.pone.0302944)
Supplement: Attachment — Submitted filename: Revision notes (R2).docx [file pone.0302944.s002.docx]

**REVISION NOTES**

Manuscript number: *PONE-D-23-41172R1*

Title:
 “*Non-destructive test-based assessment of uniaxial compressive strength and elasticity modulus of intact carbonate rocks using stacking ensemble models”*

April 10, 2024

Dear Editor,

Thank you and the reviewers very much for your kindness and useful comments and suggestions on the manuscript. After carefully read them, we followed your recommendations and modified the manuscript accordingly. The detailed responses to all of the comments are listed below point by point. The performed corrections are marked by red color in the file of “Manuscript marked-up Version (R2)”. I would be grateful if you would examine the possibility of this paper for publication. I look forward to hear from you as soon as possible. Please do not hesitate to contact me for any more clarifications.

Sincerely yours

Corresponding author

Response to the comments of Editor

**Editor Comments:**

**Journal Requirements:**

Please review your reference list to ensure that it is complete and correct. If you have cited papers that have been retracted, please include the rationale for doing so in the manuscript text, or remove these references and replace them with relevant current references. Any changes to the reference list should be mentioned in the rebuttal letter that accompanies your revised manuscript. If you need to cite a retracted article, indicate the article’s retracted status in the References list and also include a citation and full reference for the retraction notice.

**Reply;** Thank you. The manuscript was reviewed for the items mentioned. There are no potential issues that apply to the manuscript.

**Additional Editor Comments:**

Hi, Davood Fereidooni, my opinion is to accept after minor revision.

**Reply;** Thank you for your kindness.

In order to make the reader clear about your conclusion, please list them.

**Reply;** Thank you for your good comment. The conclusion section was presented in the form of numbered paragraphs.

In addition, many of the images in the manuscript are not clear, please replace them with vector graphics.

**Reply;** Thank you for you good comment. I did not understand which images are not clear. Also, the images cannot be rendered with vector graphics. Anyway, I checked all the images and increased quality of some images if needed.

Please delete at least 6 references from references 38-47.

**Reply;** Thank you. Seven references were deleted from the list and they removed from the main text of the manuscript. All required correction were done.

Finally, the authors would like to thank for your kindly suggestions through the manuscript.

Best regards

Corresponding Author
